# Pathophysiological and immunological characterization of a murine excisional wound infection model for cutaneous *Mycobacterium abscessus* infection

**DOI:** 10.3389/fcimb.2026.1828052

**Published:** 2026-07-31

**Authors:** Yuri Jung, Connor Wood, Joong-Yub Kim, Nakwon Kwak, Taek Soo Kim, Jae-Joon Yim, Siyeon Yang

**Affiliations:** 1 Animal Research and Operation Laboratory, Institut Pasteur Korea, Seongnam, Republic of Korea; 2 Antibacterial Resistance Laboratory, Institut Pasteur Korea, Seongnam, Republic of Korea; 3 Division of Pulmonary and Critical Care Medicine, Seoul National University College of Medicine, Seoul, Republic of Korea; 4 Department of Laboratory Medicine, Seoul National University Hospital, Seoul, Republic of Korea; 5 Department of Internal Medicine, Seoul National University College of Medicine, Seoul, Republic of Korea; 6 Department of Advanced Drug discovery & development, University of Science and Technology (UST), Daejeon, Republic of Korea

**Keywords:** cutaneous infection model, *Mycobacterium abscessus*, nontuberculous mycobacteria, Th1 immune response, virulence potential

## Abstract

**Introduction:**

*Mycobacterium abscessus* is an emerging rapidly growing nontuberculous mycobacterium that causes chronic skin and soft tissue infections, but its pathogenic potential remains difficult to assess in vivo because existing animal models do not fully recapitulate localized cutaneous infection.

**Methods:**

We established a murine excisional wound infection model designed to mimic cutaneous barrier disruption and compared it with conventional subcutaneous inoculation. BALB/c mice were infected with a rough-morphotype clinical isolate or rough-morphotype ATCC 19977, and lesion development, bacterial recovery, histopathology, local cytokine mRNA expression, and serum cytokine/chemokine profiles were assessed at terminal time points.

**Results:**

Compared with subcutaneous injection, the wound model generated persistent lesions with histopathological features consistent with pyogranulomatous inflammation, including foamy macrophages, multinucleated giant cells, fibroblast proliferation, and localized interferon-gamma (IFNgamma) expression. In the strains tested, the model revealed strain-dependent differences in infection establishment: the clinical isolate produced frequent chronic nodular lesions, whereas roughmorphotype ATCC 19977 showed lower lesion incidence under the same experimental conditions. Local tissue and serum cytokine profiling demonstrated a T helper 1 (Th1)-skewed immune response detectable at both terminal time points, associated with bacterial recovery from infected tissues and detectable splenic bacterial recovery.

**Discussion:**

These findings suggest that the excisional wound model provides a clinically relevant platform for studying localized cutaneous *M. abscessus* infection. Further validation using additional clinical isolates and mechanistic studies will be required to define its broader applicability.

## Introduction

*Mycobacterium abscessus (M. abscessus)* is a rapidly growing mycobacterium (RGM) and a formidable pathogen responsible for an increasing number of nontuberculous mycobacteria (NTM) infections worldwide (Griffith et al., 2007; Adjemian et al., 2012). While often associated with chronic pulmonary diseases, *M. abscessus* is also a major cause of severe skin and soft tissue infections (SSTIs) following trauma, surgery, or cosmetic procedures (Uslan et al., 2006; Lee et al., 2010). These cutaneous infections are notoriously difficult to treat due to the bacterium’s intrinsic multi-drug resistance and propensity to form biofilms, frequently leading to persistent abscesses, non-healing ulcers, and disseminated disease (Nessar et al., 2012). Despite the clinical urgency, the precise pathophysiological mechanisms governing host-pathogen interactions in cutaneous *M. abscessus* infections remain incompletely understood.

The development of effective therapeutic strategies is currently hampered by the lack of animal models that effectively mimic human cutaneous infection. While zebrafish embryos and intravenous mouse models have provided valuable insights into systemic dissemination and granuloma formation, they do not replicate the microenvironment of localized skin infection (Ordway et al., 2008; Bernut et al., 2014). Subcutaneous (S.C.) injection models are widely used for drug screening; however, they rely on the artificial administration of high bacterial loads into the subcutaneous space. This method often induces a nonspecific acute inflammatory response and abscess formation driven by the inoculum size rather than the intrinsic virulence of the pathogen, failing to recapitulate the complex histopathological features, such as organized granulomas, observed in human patients (Obregón-Henao et al., 2015; Basaraba and Hunter, 2017).

A critical limitation of existing models is their inability to discriminate the subtle heterogeneity in virulence among *M. abscessus* variants. *M. abscessus* exhibits two distinct colony morphotypes: smooth (S) and rough (R), with the R-morphotype generally linked to increased virulence and intracellular survival (Howard et al., 2006). However, virulence heterogeneity exists even within the R-morphotype. Laboratory-adapted strains, such as ATCC 19977, may undergo attenuation following prolonged *in vitro* subculturing, whereas clinical isolates often retain robust virulence factors required to evade host immunity (Byrd and Lyons, 1999; Catherinot et al., 2007). Current infection models frequently fail to distinguish between these “attenuated” and “hyper-virulent” phenotypes, obscuring the evaluation of true pathogenic potential.

In this study, we established a murine excisional wound infection model to bridge the gap between artificial inoculation and clinically relevant cutaneous infection. Unlike the S.C. injection route, the wound model mimics bacterial entry through disrupted skin barriers. We tested whether this model could reproduce key histopathological and immunological features of cutaneous *M. abscessus* infection and whether it could reveal strain-dependent differences in infection establishment. Using a rough-morphotype clinical isolate and a rough-morphotype ATCC 19977 strain, we observed differential nodular lesion formation and distinct local and systemic immune responses. These findings support the wound model as a physiologically relevant platform for studying cutaneous *M. abscessus* infection, while further validation with additional clinical isolates will be required to determine its broader utility for evaluating strain-dependent pathogenic phenotypes.

## Materials and methods

### Bacterial strains and growth conditions

*M. abscessus* ATCC 19977 was obtained from the American Type Culture Collection (ATCC) and used for all experiments. A clinical isolate of *M. abscessus*, originally recovered from a patient with pulmonary infection, was obtained from Seoul National University Hospital (SNUH). ATCC 19977 initially presented as a mixed smooth- and rough-morphotype culture; therefore, a uniform rough-morphotype culture was established from an individual rough colony isolated on Middlebrook 7H10 agar. The SNUH clinical isolate presented as a uniform rough-morphotype culture, and stocks were prepared from a single isolated colony (**Supplementary Figure 1**).

*M. abscessus* strains were grown at 37°C under shaking at 100 rpm in Middlebrook 7H9 broth (Difco, USA) supplemented with 0.2% (vol/vol) glycerol, 0.05% Tween 80, and 10% (vol/vol) oleic acid-albumin-dextrose-catalase (OADC; Becton, Dickinson and Company, USA). Bacterial growth was monitored using a spectrophotometer at an optical density of 600 nm (OD600).

### Mice

Specific-pathogen-free (SPF) female BALB/c mice (6 weeks old) were purchased from Orient Bio Inc. (Seongnam, Korea). Mice were maintained in Biosafety Level 2 (BSL-2) facilities at Institut Pasteur Korea and provided with sterile water, standard chow, and bedding ad libitum. Mice were acclimatized in the facility for one week prior to the experiments; thus, the mice were 7 weeks old at the start of infection. All animal studies were approved by the Institutional Animal Care and Use Committee (IACUC) of Institut Pasteur Korea in strict accordance with the Korean Animal Protection Law (Protocol # IPK-24003).

### Murine skin infection model

At each terminal time point, mice were randomly allocated to the non-infected control group (n = 5 per time point) or the infected group (n = 7 per route and time point). Separate cohorts of mice were euthanized at days post-infection (DPI) 21 and DPI 28 for terminal analyses, including colony-forming unit (CFU) enumeration, histopathology, reverse transcription quantitative PCR (RT-qPCR), and serum cytokine profiling. Therefore, all reported n values indicate the number of mice per group at each time point, not the total number of animals across the entire study. Each mouse was considered an independent biological replicate.

Mice were anesthetized via intraperitoneal injection of a mixture of ketamine (100 mg/kg; Yuhan Ketamine 50 Inj., Yuhan Corporation, Seoul, Korea) and xylazine (4 mg/kg; Rompun 2%, Elanco Animal Health Korea Co., Ansan, Korea). The intrascapular region was shaved using electric clippers prior to infection. The wound infection model was established by creating three parallel 1.5 cm full-thickness incisions, followed by inoculation of the incisions with 50 µL of bacterial suspension containing 5 × 10^6^ CFU per mouse. Control mice received 50 µL of phosphate-buffered saline (PBS). For the subcutaneous infection model, mice received the same inoculum dose, 5 × 10^6^ CFU in 50 µL, by subcutaneous injection. Mice were monitored daily for signs of skin infection and distress and were euthanized on days 21 and 28. The wound area size was measured using a caliper at 4-day intervals, and the lesion area (mm²) was calculated by multiplying the width (mm) by the length (mm) of each lesion. Photographic documentation was also performed at each time point. For the determination of bacterial load, skin lesion tissues were aseptically excised using an 8-mm punch biopsy and homogenized in 2 mL of sterile 1 × PBS using a gentleMACS™ Dissociator (Miltenyi Biotec, Bergisch Gladbach, Germany). The homogenates were serially diluted and plated onto Middlebrook 7H11 agar plates (Sigma-Aldrich, St. Louis, MO, USA) supplemented with 10% OADC enrichment (Becton Dickinson) and 10 μg/mL amphotericin B (Sigma-Aldrich). *M. abscessus* colonies were enumerated after 4 days of incubation at 37 °C. Full-thickness biopsies of the central cutaneous lesions were collected, fixed in 10% neutral buffered formalin (4% formaldehyde; Sigma-Aldrich), and processed for histopathological examination.

### Histological analysis

Fixed tissues were processed, embedded in paraffin, and sectioned at a thickness of 4 µm. For histopathological evaluation, tissue sections were stained with hematoxylin and eosin (H&E), acid-fast bacilli (AFB) stain using the Ziehl–Neelsen method, or processed for immunohistochemistry (IHC). All staining procedures were performed according to standard protocols, and stained sections were examined under a light microscope (Axioscope 5, Zeiss, Oberkochen, Germany).

H&E staining was performed using the NovaUltra™ H&E Stain Kit (Rockville, MD, USA). Slides were deparaffinized in xylene (3 changes, 5 min each) and rehydrated through graded alcohols (100% for 5 min, 95% and 70% for 2 min each). Slides were stained with Mayer’s hematoxylin for 2 min, washed with running tap water for 2 min, rinsed in 95% alcohol, counterstained with eosin for 45 s, and rinsed again in 95% alcohol. Finally, slides were dehydrated, cleared in xylene, and mounted with a xylene-based mounting medium (Vector Laboratories, Burlingame, CA, USA).

AFB detection was performed after deparaffinization and rehydration as described above using the YD Diagnostics AFB Stain Kit (Youngin, Korea). Sections were flooded with carbol fuchsin and heated gently until steaming for 5 min. After rinsing with distilled water, sections were decolorized with 1% acid alcohol for 2 min, washed, and counterstained with 0.1% methylene blue for 30 s to 1 min. Acid-fast bacilli appeared as bright red rods against a blue background. The bacillary load was evaluated using a modified Ridley’s Index (Mitchell et al., 2022). AFB-positive bacilli were counted in at least three randomly selected fields per sample.

IHC analysis was performed on deparaffinized tissue sections. Antigen retrieval was carried out by heating the sections in citrate buffer (10 mM, pH 6.0) at 90-100 °C for 20 min. Endogenous peroxidase activity was blocked with 3% hydrogen peroxide for 10 min at room temperature. Sections were blocked with normal serum for 1 h at room temperature to reduce nonspecific binding and incubated with an anti–IFN-γ primary antibody (PA5-95560, Invitrogen, Carlsbad, CA, USA) at a dilution of 1:1000 overnight at 4 °C, followed by incubation with a horseradish peroxidase (HRP)-conjugated secondary antibody (Vector Laboratories) for 1 h at room temperature, and a streptavidin–HRP conjugate (Vectastain ABC kit; Vector Laboratories) according to the manufacturer’s instructions. Staining was visualized using a 3,3’-diaminobenzidine (DAB) chromogen, and nuclei were counterstained with hematoxylin. The IFN-γ-positive area (%) was quantified using ImageJ software (NIH, Bethesda, MD, USA) by calculating the ratio of DAB-positive pixels to the total tissue area.

### Cytokine assay

Blood was collected via cardiac puncture under anesthesia. Blood samples were allowed to clot at room temperature for 30 min and centrifuged at 1,500 × g for 10 min at 4 °C. Serum was collected and stored at −80 °C until analysis. Cytokine and chemokine levels were measured using a Luminex Mouse High Sensitivity T Cell Panel 9-plex (Merck Millipore, Burlington, MA, USA; Cat# MIL-MHSTCMAG-70K09), targeting IFN-γ, IL-1β, IL-2, IL-6, IL-10, IL-12(p70), IL-17A, tumor necrosis factor-α (TNF-α), and macrophage inflammatory protein-2 (MIP-2). The assay was performed using a Luminex 100/200™ analyzer (Luminex Corp., Austin, TX, USA) through LABIS KOMA Inc. (Seoul, Korea), following the manufacturer’s instructions.

### RNA extraction and reverse transcription quantitative PCR (RT-qPCR)

Total RNA was extracted from the skin tissues of *M. abscessus* infected mice using TRIzol reagent (Invitrogen, USA) according to the manufacturer’s instructions. RNA purity and concentration were measured using a NanoVue spectrophotometer (GE Healthcare Life Sciences, USA). Complementary DNA (cDNA) was synthesized from 1 μg of total RNA using a reverse transcription kit (Takara, Japan). Quantitative real-time PCR was performed on a QuantStudio 5 Real-Time PCR System (Thermo Fisher Scientific, USA) with SYBR Green dye (Applied Biosystems, USA). The primers were synthesized by Genotech (Korea), and the sequences used for *RpoB*, ITS, IFN-γ, IL-6, and IL-10 are listed in **Supplementary Table 1**. The expression levels of NTM-specific marker genes (*RpoB* and *ITS*) were determined based on their Ct values. Relative mRNA expression levels of cytokines (IFN-γ, IL-6, and IL-10) were quantified using the 2^−ΔΔCt method and normalized to 18S rRNA as an internal control.

### Statistical analysis

**Figure 1:**
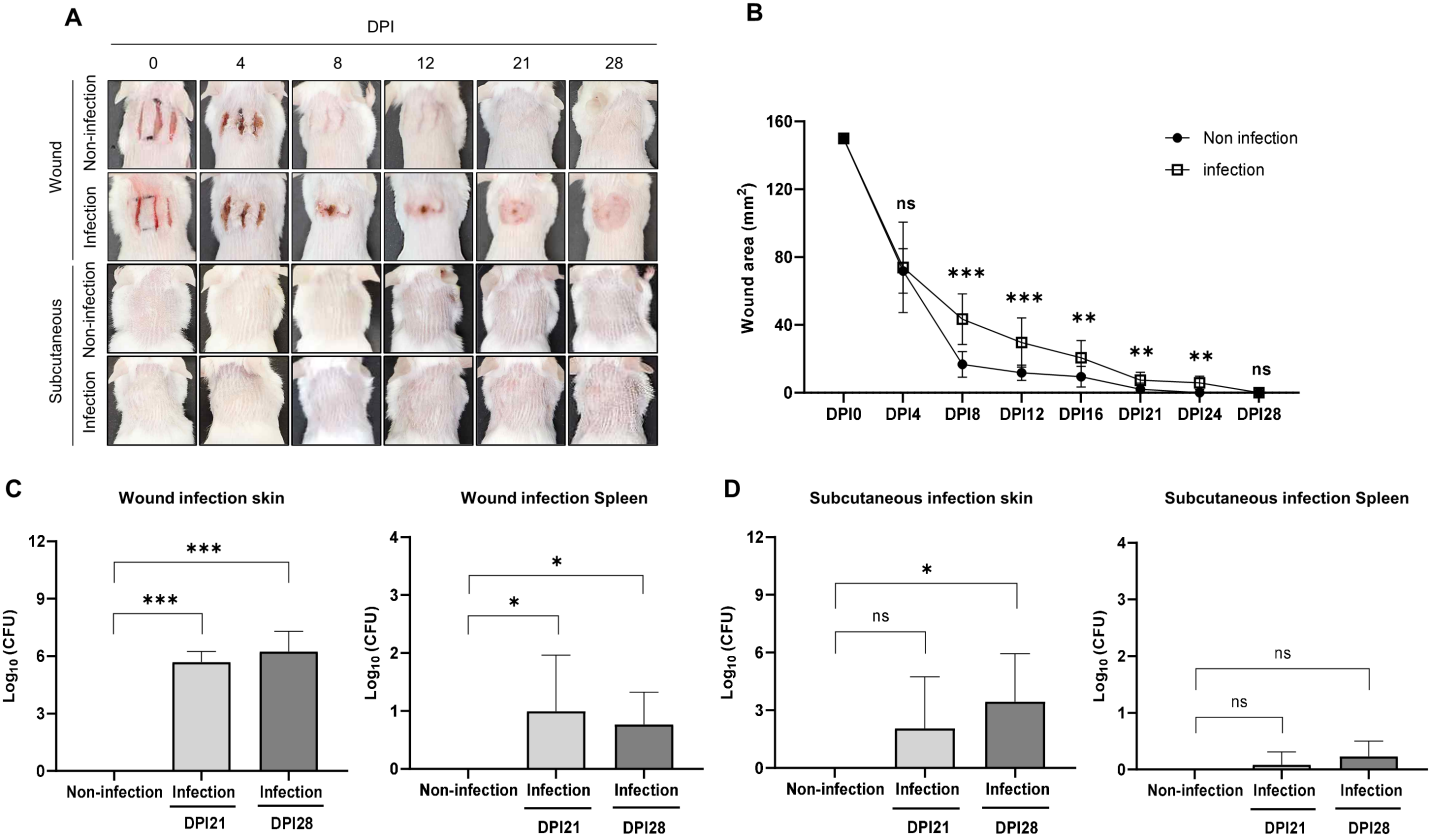
Establishment of a murine cutaneous *M. abscessus* infection model by excisional wound inoculation. Three parallel 1.5 cm full-thickness incisions on the intrascapular skin of BALB/c mice were inoculated with 50 μL of 5 × 10^6 CFU rough-morphotype clinical isolate or PBS (non-infected control); the subcutaneous (S.C.) group received the same dose by injection. **(A)** Representative skin lesion photographs at the indicated time points. **(B)** Lesion area in the wound model, measured longitudinally in the same mice at four-day intervals; analyzed by linear mixed-effects model (mouse as random effect; infection status × time as fixed effects) with Sidak post-hoc at each time point. **(C, D)** Skin and spleen bacterial burdens in the wound **(C)** and subcutaneous **(D)** models. Within each tissue, the three groups (non-infected, infected DPI 21, infected DPI 28) were compared on log10-transformed CFU by one-way ANOVA followed by Tukey's HSD. n = 5 non-infected and n = 7 infected mice per route per time point; separate cohorts were euthanized at DPI 21 and DPI 28 for panels **(C, D)** Bars indicate mean ± SEM with individual data points overlaid. ns, not significant; *p < 0.05; **p < 0.01; ***p < 0.001.

**Figure 2:**
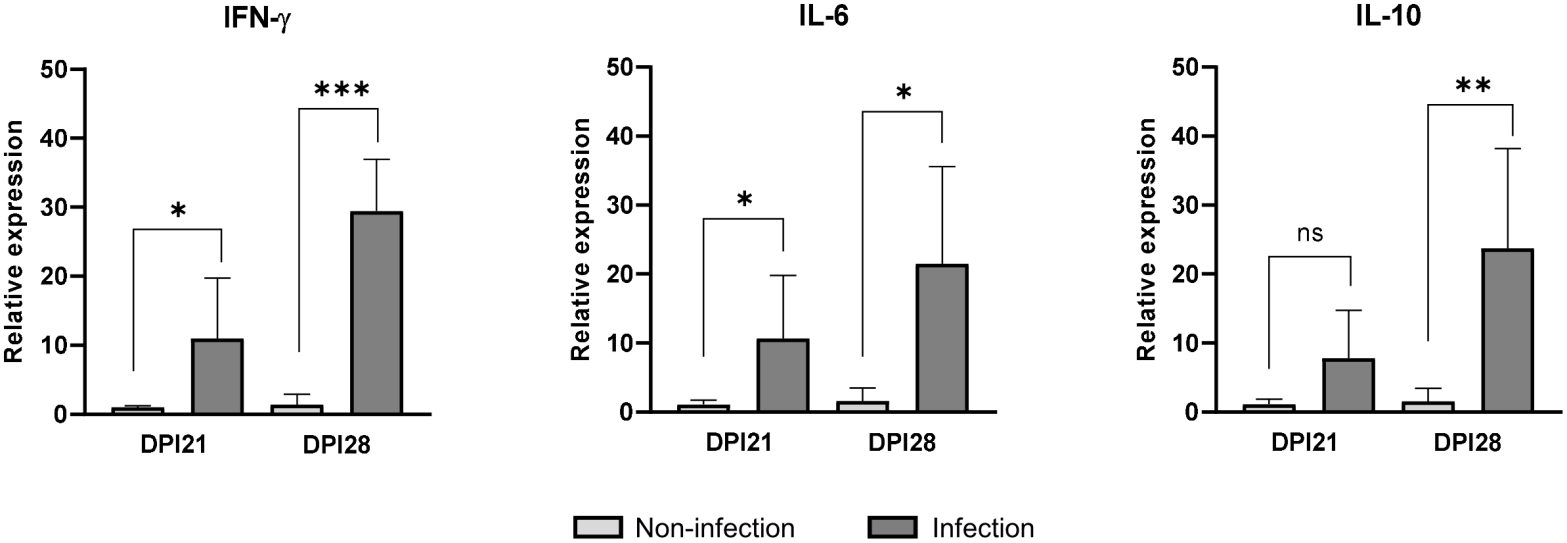
Local cytokine mRNA expression in skin tissue during *M. abscessus* wound infection. Relative mRNA expression of IFN-γ, IL-6, and IL-10 was quantified by RT-qPCR and normalized to 18S rRNA. Non-infected (n = 5) and infected (n = 7) mice were analyzed at DPI 21 and DPI 28 using separate cohorts per time point. Bars indicate mean ± SEM with individual data points overlaid. Statistical significance was determined by two-tailed Welch's t-test comparing non-infected versus infected groups at each time point; the design did not include longitudinal comparison across time points. ns, not significant; *p < 0.05; **p < 0.01; ***p < 0.001.

**Figure 3:**
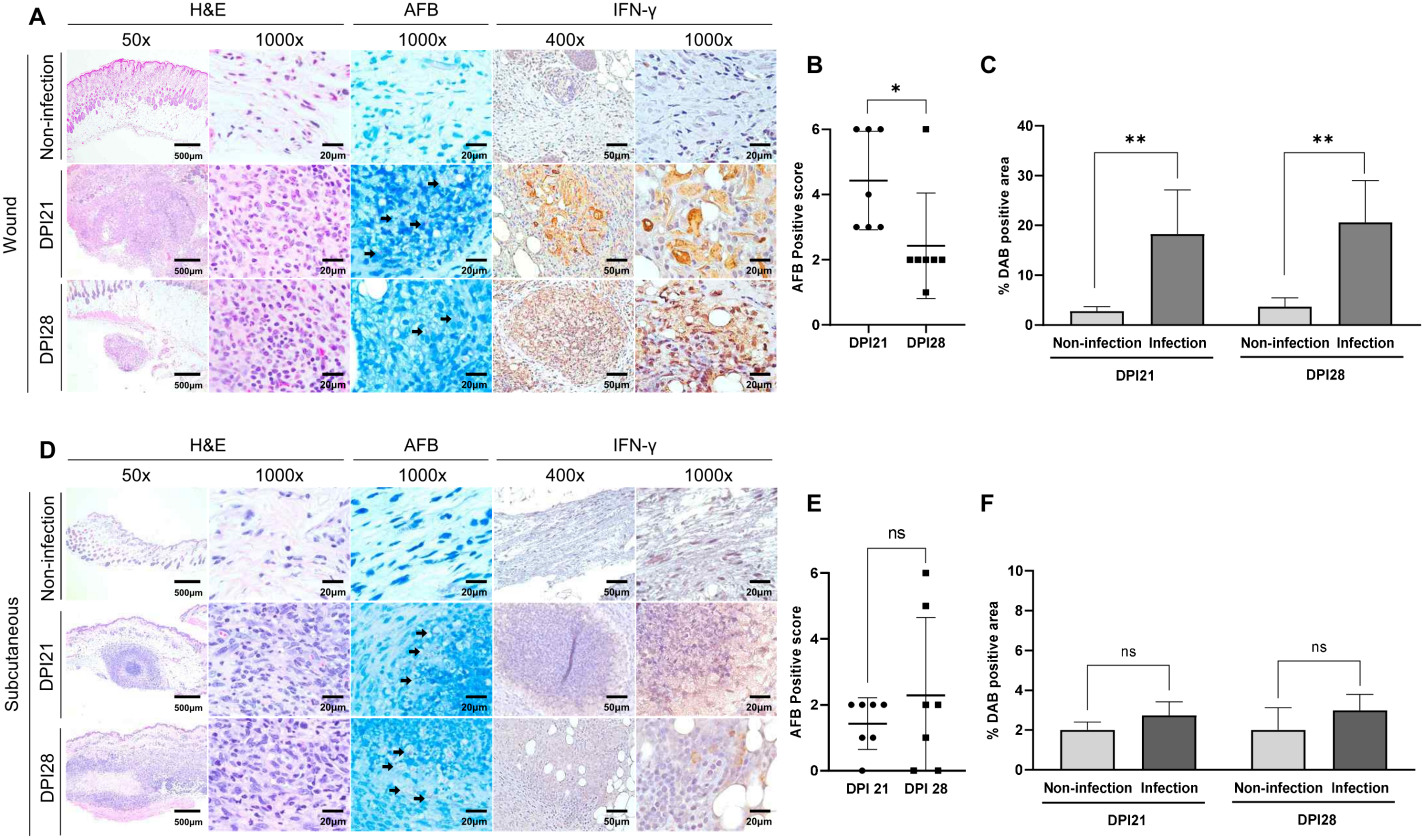
Histopathological features and IFN-γ localization in wound and subcutaneous *M. abscessus* lesions. **(A, D)** Representative H&E, AFB, and IFN-γ IHC images from the wound **(A)** and subcutaneous **(D)** infection models at DPI 21 and DPI 28. **(B, E)** AFB-positive bacillary scores (modified Ridley's index) in wound **(B)** and subcutaneous **(E)** lesions; DPI 21 versus DPI 28 compared within each route by Mann-Whitney U test. **(C, F)** IFN-γ-positive area (% DAB-positive, ImageJ) in wound **(C)** and subcutaneous **(F)** lesions; non-infected versus infected compared within each time point by Welch's t-test. n = 5 non-infected and n = 7 infected mice per time point per route; separate cohorts at each terminal time point. Bars indicate mean ± SEM with individual data points overlaid. Representative images were obtained at 50×, 400×, and 1000× magnifications. Arrows indicate representative acid-fast bacilli in high-magnification AFB images. Scale bars: 500 μm for 50× images, 50 μm for 400× images, and 20 μm for 1000× images. ns, not significant; *p < 0.05; **p < 0.01.

**Figure 4:**
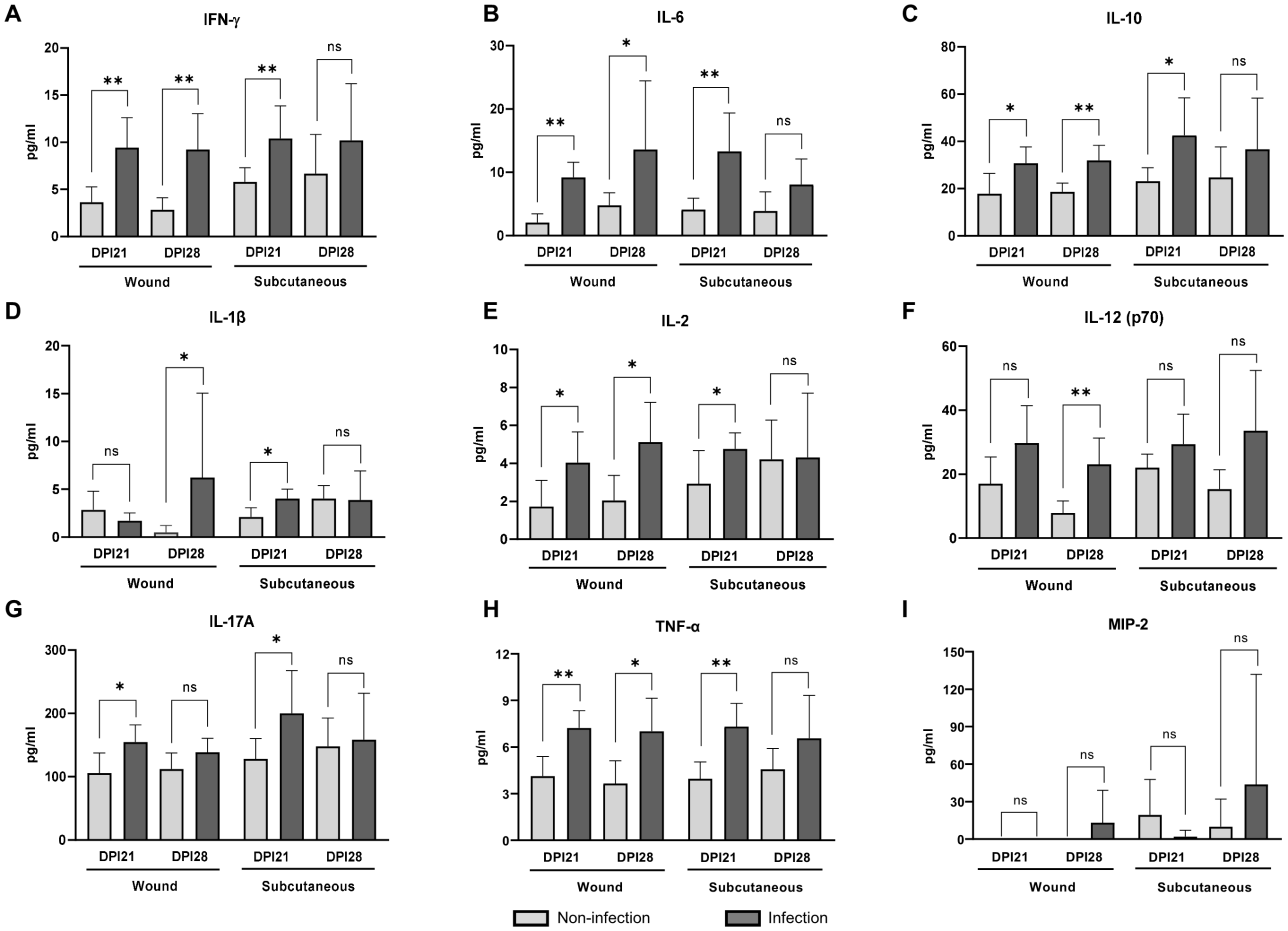
Serum cytokine and chemokine profiles in the wound and subcutaneous *M. abscessus* infection models. Serum concentrations of **(A)** IFN-γ, **(B)** IL-6, **(C)** IL-10, **(D)** IL-1β, **(E)** IL-2, **(F)** IL-12(p70), **(G)** IL-17A, **(H)** TNF-α, and **(I)** MIP-2 were measured at DPI 21 and DPI 28 by multiplex Luminex assay. Within each route and time point, non-infected and infected groups were compared by two-tailed Mann-Whitney U test; no statistical comparison was performed across time points or between routes. n = 5 non-infected and n = 7 infected mice per route per time point; separate cohorts were euthanized at each terminal time point. Bars indicate mean ± SEM with individual data points overlaid. ns, not significant; *p < 0.05; **p < 0.01.

All data are presented as mean ± standard error of the mean (SEM) with individual data points overlaid where applicable. Statistical analyses were performed using GraphPad Prism (version 9.0; GraphPad Software, San Diego, CA, USA) and R (version 4.x). Wound area (**Figure 1B**), measured longitudinally in the same animals, was analyzed by a linear mixed-effects model with infection status and time as fixed effects and individual mouse as a random effect, followed by Sidak-adjusted *post-hoc* comparisons at each time point. Skin and spleen bacterial burdens (**Figures 1C, D**) were log10-transformed and compared among the three groups (non-infected, infected DPI 21, infected DPI 28) within each tissue by one-way ANOVA followed by Tukey’s HSD. Local cytokine mRNA expression (**Figure 2**) and IFN-γ-positive area (**Figures 3C, F**) were compared between non-infected and infected groups within each time point by two-tailed Welch’s t-test to accommodate unequal group sizes (n = 5 vs 7) and unequal variances. AFB-positive bacillary scores (**Figures 3B, E**), a semi-quantitative ordinal index, were compared between time points within each route by the Mann-Whitney U test. Serum cytokine concentrations (**Figure 4**) were compared between non-infected and infected groups within each route and time point by the Mann-Whitney U test, using exact p-values where possible and the normal approximation with continuity correction in the presence of ties. No statistical comparison was performed across time points or between infection routes. The statistical test used for each dataset is indicated in the corresponding figure legend. A two-sided p-value < 0.05 was considered statistically significant. ns, not significant; *p < 0.05; **p < 0.01; ***p < 0.001.

## Results

### Differential nodular lesion formation according to bacterial strain and cutaneous infection route

To determine whether *M. abscessus* can establish productive cutaneous infection in mice and generate nodular lesions, ATCC 19977 and a clinical isolate were inoculated via either the S.C. injection route or the excisional wound route. Lesion development was evaluated at DPI 21 and DPI 28.

In the wound infection model, mice infected with the clinical isolate showed nodular lesion formation in 100% of animals at DPI 21 (7/7) and 71.4% at DPI 28 (5/7). In contrast, wound infection with ATCC 19977 resulted in nodular lesion formation in 14.3% of mice at DPI 21 (1/7) and 28.6% at DPI 28 (2/7). In the S.C. model, the clinical isolate produced nodular lesions in 28.6% of mice at both DPI 21 and DPI 28 (2/7 at each time point), whereas ATCC 19977 produced nodular lesions in 0% of mice at DPI 21 (0/7) and 14.3% at DPI 28 (1/7) (**Table 1**).

**Table 1:** Nodule incidence in BALB/c mice infected with *M. abscessus* ATCC 19977 or the clinical isolate through wound or subcutaneous routes.

Strain	Infection route	DPI	Nodule incidence % (no.)
ATCC 19977	Wound	DPI 21	14.3 (1/7)
		DPI 28	28.6 (2/7)
ATCC 19977	Subcutaneous	DPI 21	0.00 (0/7)
		DPI 28	14.3 (1/7)
Clinical	Wound	DPI 21	100 (7/7)
		DPI 28	71.4 (5/7)
Clinical	Subcutaneous	DPI 21	28.6 (2/7)
		DPI 28	28.6 (2/7)

DPI, days post-infection, n = 7 per strain-route-time point.

Both strains displayed a rough colony morphotype. In broth culture, ATCC 19977 showed faster growth and reached a higher maximum population density than the clinical isolate (**Supplementary Figure 1**). The corresponding *in vivo* nodule incidence for each strain and infection route is summarized in **Table 1**.

### Establishment of a chronic cutaneous *M. abscessus* infection model via excisional wounding

Wound area was monitored longitudinally in the same animals at four-day intervals throughout the observation period. Linear mixed-effects modeling, with infection status and time as fixed effects and mouse as a random effect, identified a significant infection × time interaction (p < 0.001) for wound area in the excisional wound model. Sidak-adjusted *post-hoc* comparisons at each time point indicated significantly larger lesion areas in infected versus non-infected mice from DPI 8 through DPI 24 (p < 0.01 at each time point), with both groups approaching baseline closure by DPI 28 (**Figure 1B**). These findings indicate that *M. abscessus* infection delayed normal wound resolution in the excisional model. Notably, the reduction in gross surface wound area by DPI 28 reflected macroscopic epithelial closure rather than complete resolution of the underlying lesion. Histological analysis showed persistent pyogranulomatous inflammation in deeper dermal and subcutaneous compartments despite apparent surface-level healing.

Bacterial viability within the lesions was confirmed by CFU enumeration, with each route analyzed independently. In the wound infection model, skin tissue CFU values were significantly elevated in both the DPI 21 (p < 0.001) and DPI 28 (p < 0.001) infected groups compared with non-infected controls, with no significant difference between the two infected time points, indicating sustained local colonization across the observation window (**Figure 1C**). Splenic CFU values in the wound model were also significantly elevated at DPI 21 (p < 0.05) and DPI 28 (p < 0.05) compared with non-infected controls, indicating detectable systemic bacterial recovery. In the subcutaneous infection model, skin tissue CFU values reached statistical significance only at DPI 28 (p < 0.05), while DPI 21 did not differ significantly from non-infected controls; splenic CFU values showed no significant elevation at either time point (**Figure 1D**). Because the present design analyzed each route independently, no direct statistical comparison of bacterial burden between the wound and subcutaneous routes was performed. The qualitative patterns observed across the two models are consistent with persistent local colonization in the wound model and a less consistent bacterial recovery profile in the subcutaneous model.

### Molecular confirmation of localized infection and skin tissue cytokine responses

To verify persistent *M. abscessus* detection within the lesions, RT-qPCR was performed using nucleic acids extracted from infected skin tissues. Amplification of NTM-specific marker genes, including *RpoB* and *ITS*, yielded consistent positive cycle threshold (Ct) values at both DPI 21 and DPI 28, supporting persistent detection of *M. abscessus* in the local tissue (**Table 2**).

**Table 2:** Representative cycle threshold values of NTM-specific marker genes in infected skin tissues from the wound infection model.

Gene	RpoB			ITS		
DPI	Ct	SD	Result	Ct	SD	Result
DPI 21	30.48	1.40	NTM positive	31.21	0.50	NTM positive
DPI 28	29.14	1.31	NTM positive	31.09	0.17	NTM positive

Skin tissues were collected from mice infected with the rough-morphotype clinical isolate via the excisional wound route at DPI 21 and DPI 28. Ct, cycle threshold; DPI, days post-infection; RT-qPCR, reverse transcription quantitative PCR; RpoB, RNA polymerase β-subunit gene; ITS, internal transcribed spacer; SD, standard deviation.

To further understand the localized immune microenvironment driven by this persistent colonization, we evaluated the relative mRNA expression of key cytokines directly from the infected skin tissues (**Figure 2**). Local cytokine expression was assessed at two independent terminal time points, with infected and non-infected tissues compared within each time point. At DPI 21, infected skin tissue showed significantly elevated IFN-γ (p < 0.05) and IL-6 (p < 0.05) compared with non-infected controls, while IL-10 showed no significant difference. At DPI 28, infected tissue likewise exhibited elevated IFN-γ (p < 0.001), IL-6 (p < 0.05), and IL-10 (p < 0.01) compared with non-infected controls. These results indicate that a Th1-skewed cytokine signature was detectable in infected tissue at both terminal time points examined, with IL-10 also reaching statistical significance at the later time point. Because the present design analyzed each time point independently, we did not test for time-dependent changes in cytokine expression within the infected group; longitudinal analysis of this dynamic will require sequential sampling within individual animals.

### The wound infection model recapitulates clinical granulomatous pathology distinct from subcutaneous abscesses

Histopathological examination of H&E- and AFB-stained skin biopsies revealed distinct tissue architectures between the wound and subcutaneous infection models. In the wound model, lesions showed pyogranulomatous inflammation characterized by neutrophils, foamy macrophages, lymphocytes, fibroblast proliferation, central necrosis, and multinucleated giant cells within deeper dermal and subcutaneous compartments (**Figure 3A**). AFB staining confirmed the presence of acid-fast bacilli within cellular aggregates, and high-magnification AFB images further demonstrated representative bacilli within the lesions. The AFB-positive bacillary score decreased significantly from DPI 21 to DPI 28 in the wound model (**Figure 3B**; p = 0.017, Mann–Whitney U test). IFN-γ IHC showed significantly increased IFN-γ-positive area in infected wound tissues compared with non-infected controls at both DPI 21 and DPI 28 (**Figure 3C**).

In contrast, the subcutaneous model showed a predominantly suppurative abscess-like pattern with extensive necrosis and neutrophil-rich inflammation, without the same degree of organized pyogranulomatous architecture observed in the wound model (**Figure 3D**). AFB-positive bacillary scores did not significantly change between DPI 21 and DPI 28 in the subcutaneous model (**Figure 3E**), and IFN-γ-positive area did not reach statistical significance at either time point (**Figure 3F**). Because each route was analyzed independently, no direct statistical comparison between routes was performed.

### Systemic immune profiling: distinct Th1 polarization in the wound model

Systemic immune responses in the two infection models were profiled by measuring nine serum cytokines and chemokines at DPI 21 and DPI 28 (**Figure 4**). Within each route and time point, infected mice were compared with non-infected controls by Mann-Whitney U test; no statistical comparison was performed across time points or between routes.

In the wound infection model, infected mice showed significantly elevated IFN-γ at both DPI 21 and DPI 28 (p < 0.01 for each), IL-6 at both DPI 21 (p < 0.01) and DPI 28 (p < 0.05), IL-10 at both DPI 21 (p < 0.05) and DPI 28 (p < 0.01), IL-2 at both DPI 21 and DPI 28 (p < 0.05 for each), and TNF-α at both DPI 21 (p < 0.01) and DPI 28 (p < 0.05) relative to non-infected controls. IL-12(p70) was significantly elevated at DPI 28 (p < 0.01) but not at DPI 21, whereas IL-17A was significantly elevated at DPI 21 (p < 0.05) but not at DPI 28. IL-1β reached significance only at DPI 28 (p < 0.05).

In the subcutaneous infection model, infected mice at DPI 21 showed significant elevation of IFN-γ (p < 0.01), IL-6 (p < 0.01), IL-10 (p < 0.05), IL-1β (p < 0.05), IL-17A (p < 0.05), and TNF-α (p < 0.01) relative to non-infected controls. At DPI 28, none of the measured cytokines reached statistical significance in the subcutaneous model. IL-12(p70), and MIP-2 showed no significant elevation in the subcutaneous model, and MIP-2 did not reach significance in either model at either time point.

Because each combination of infection route and time point was analyzed independently, formal statistical comparison across time points or between routes was not performed. In the wound model, IFN-γ, IL-10, IL-2, and TNF-α reached significance at both terminal time points, accompanied by delayed elevation of IL-12(p70) at DPI 28; this pattern is consistent with engagement of a Th1-associated cytokine signature together with the immunoregulatory cytokine IL-10. In the subcutaneous model, all detected cytokine elevations were confined to the earlier time point, consistent with a predominantly early systemic response.

## Discussion

The primary objective of this study was to overcome the limitations of existing animal models by establishing a murine excisional wound infection model that recapitulates the pathophysiology of human cutaneous *M. abscessus* infection. Establishing a persistent infection model for *M. abscessus* in immunocompetent mice has historically proven challenging (Rimal et al., 2025). Unlike Mycobacterium tuberculosis, which readily establishes chronic pulmonary infection, *M. abscessus* is frequently characterized by rapid spontaneous clearance in murine respiratory models due to efficient innate immune clearance mechanisms, as demonstrated by Ordway et al. (2008). This transient nature of infection in standard models complicates the evaluation of long-term pathogenesis and therapeutic efficacy. Furthermore, while S.C. injection models have been widely utilized for drug screening, their reliance on artificial administration of high bacterial loads into the subcutaneous space frequently results in acute suppurative abscesses rather than the chronic granulomatous lesions observed in clinical settings (Ordway et al., 2008; Obregón-Henao et al., 2015). In clinical settings, cutaneous *M. abscessus* infections are distinguished by the formation of specific erythematous nodules and granulomas rather than simple non-specific abscesses (Lee et al., 2010). Reproducing this nodular phenotype is therefore critical for the translational value of any cutaneous NTM model.

In the present investigation, comparison of the S.C. and wound infection models revealed distinct pathological trajectories. The S.C. model was characterized by acute inflammation, extensive necrosis, and neutrophil dominance, lacking the organized structural integrity of chronic infection. The wound infection model, in contrast, overcame the tendency for spontaneous clearance observed in respiratory models, demonstrating the formation of organized granulomas containing multinucleated giant cells, foamy macrophages, and fibroblast proliferation. These histopathological features closely mirror the NTM-specific nodular lesions reported in human patients (Lee et al., 2010). It is postulated that the interplay between the wound healing process and bacterial colonization creates a unique microenvironment that favors the development of chronic granulomatous inflammation, thereby providing a physiologically relevant platform that mirrors the clinical reality of nodule formation more accurately than traditional injection methods (Bohaud et al., 2022). An important consideration in interpreting this model is that excisional wounding itself initiates active tissue repair and remodeling. Therefore, some components of the lesion microenvironment, including IL-6 and IL-10 expression, macrophage accumulation, and fibroblast proliferation, may reflect the combined effects of bacterial persistence and wound-repair biology rather than infection-driven inflammation alone. This overlap is not necessarily a limitation of the model, because human cutaneous *M. abscessus* infection often develops in the context of trauma, procedures, or barrier disruption. Nevertheless, future studies incorporating uninfected wound-repair controls with extended histological and immunological profiling will be important to separate wound-healing-associated responses from pathogen-specific inflammatory mechanisms. With respect to systemic spread, splenic bacterial recovery was detectable in the wound model at DPI 21 and DPI 28, whereas splenic bacterial recovery in the S.C. model was minimal or undetectable at both time points. Because each route was analyzed independently, no direct statistical comparison between routes was performed; nonetheless, these independent observations are consistent with the possibility that wound inoculation favors detectable systemic bacterial recovery under the tested conditions. The route and cellular mechanism underlying this systemic detection were not directly examined in the present study.

Beyond histopathological fidelity, the present data suggest that the wound model may capture strain-dependent differences in cutaneous infection establishment. Although both strains examined in this study displayed a rough morphotype, the clinical isolate showed a higher incidence of nodular lesion formation than ATCC 19977 in the wound model. This difference may be related to isolate-specific pathogenic traits that are not reflected by *in vitro* growth kinetics alone. In previous studies, rough-morphotype *M. abscessus* cording was shown to impair macrophage phagocytosis and promote abscess formation in a zebrafish infection model (Bernut et al., 2014). Thus, cording-associated immune evasion may represent one possible factor contributing to the enhanced lesion formation observed with the clinical isolate. However, cording formation, macrophage uptake, and intracellular bacterial survival were not directly assessed in the present study; this mechanism therefore remains speculative and requires further experimental validation. Because the current comparison included only one clinical isolate and one laboratory-adapted strain, additional clinical isolates will be needed to determine whether the observed strain-dependent phenotype applies more broadly to clinically diverse *M. abscessus* strains.

The pathological differences observed between the wound and S.C. infection models were accompanied by distinct local and systemic immune profiles. Within the local infectious microenvironment, infected wound tissue showed significant elevation of the pro-inflammatory cytokines IFN-γ and IL-6 relative to non-infected controls at both terminal time points examined, together with elevation of the immunoregulatory cytokine IL-10 at the later time point. The detectable induction of IL-10 alongside IFN-γ is consistent with a localized, balanced immunoregulatory network within the skin, a feature associated with chronic mycobacterial containment and tissue remodeling. Because the local cytokine data were obtained from separate cohorts at each terminal time point, these findings describe the cytokine status at each time point rather than a directly tested temporal trajectory.

Serum cytokine profiling, analyzed independently within each route, revealed cytokine elevations whose composition differed between the two infection models and between the two terminal time points examined. In the wound model, IFN-γ, IL-10, IL-2, and TNF-α were significantly elevated relative to non-infected controls at both DPI 21 and DPI 28, whereas in the S.C. model all detected elevations were confined to DPI 21, with no cytokine reaching significance at DPI 28. Although the present design did not permit formal statistical comparison across time points, the cross-sectional pattern—a Th1-associated signature detectable at both time points in the wound model versus elevations restricted to the early time point in the S.C. model—parallels the organized granulomatous histopathology and local IFN-γ immunoreactivity observed in the wound lesions, and is consistent with the acute suppurative pathology characteristic of the subcutaneous abscesses.

The serum cytokine pattern further suggests that the wound model engages a more sustained and structured immune program than the S.C. model. In the wound model, IFN-γ, IL-10, IL-2, and TNF-α were significantly elevated at both terminal time points, while IL-12(p70) reached significance at DPI 28. Because IL-12 is a key cytokine produced by activated antigen-presenting cells that promotes Th1 differentiation and IFN-γ production, this late IL-12(p70) elevation may reflect sustained Th1-polarizing activity within the wound infection environment (Cooper et al., 2007). IL-17A was elevated at DPI 21 but not DPI 28, consistent with a possible early neutrophil-associated component, as IL-17A can be produced by γδ T cells and other lymphocyte populations and has been implicated in neutrophil recruitment and granuloma formation during mycobacterial infection (Lockhart et al., 2006; Okamoto Yoshida et al., 2010). IL-1β showed route- and time-dependent differences, with early elevation in the S.C. model and later elevation in the wound model, suggesting that inflammasome-associated inflammatory responses may differ between the two routes (Lee et al., 2012). However, because cytokine profiles were assessed cross-sectionally using separate cohorts at each terminal time point, these temporal interpretations remain hypothesis-generating and should be tested in future studies using longitudinal sampling, immune-cell phenotyping, and mechanistic perturbation approaches.

Taken together, these cytokine signatures are consistent with a coordinated immune program involving an early IL-17A/IL-1β-associated neutrophilic component and a later IL-12/IFN-γ-associated Th1 component, although this proposed sequence remains to be validated by longitudinal sampling. This Th1-associated signature was spatially corroborated by immunohistochemical analysis, which localized IFN-γ expression directly within the organized granulomatous lesion. Collectively, these integrated local and systemic profiles indicate that the wound model recruits and maintains an adaptive immune response. Rather than completely clearing the pathogen, this Th1-polarized environment establishes an immunological balance characteristic of chronic mycobacterial infections (Bold and Ernst, 2009), allowing the pathogen to persist within a localized granulomatous niche while permitting detectable bacterial recovery from the spleen. The detection of splenic bacteria despite a Th1-associated response raises the possibility that dissemination may occur through host-cell-associated trafficking from the disrupted skin barrier. By analogy with other pathogenic mycobacterial systems, *M. abscessus* may survive within recruited phagocytes and be transported to secondary lymphoid organs. However, the present study did not directly track infected macrophages or test macrophage dependence using macrophage depletion, lineage tracing, or intracellular survival assays. Therefore, macrophage-mediated dissemination should be considered a working hypothesis rather than a mechanism demonstrated by the current study.

Based on the observed differences in lesion incidence between ATCC 19977 and the clinical isolate, despite their shared rough morphotype, future investigations should prioritize uncovering the genetic drivers of this divergence. To this end, a comparative genomic approach using whole-genome sequencing is warranted to map single nucleotide polymorphisms and structural variations. Particular attention should be paid to candidates associated with cell envelope remodeling and stress adaptation, such as the mycobacterial membrane protein large (mmpL) gene family and the transcriptional regulator whiB7. The mmpL family is recognized for its critical role in lipid transport and cell wall architecture, directly influencing virulence and drug efflux (Dubois et al., 2018), while whiB7 acts as a master regulator of intrinsic resistance and physiological stress responses (Hurst-Hess et al., 2017). Subsequent functional validation through the generation of targeted knockout or overexpression mutants will be crucial to confirm whether manipulating these loci alters the invasive phenotype. Ultimately, this comprehensive strategy holds the potential to unveil novel virulence mechanisms and identify precise targets for drug-resistant *M. abscessus*.

This study has several limitations. First, strain-dependent differences in infection establishment were assessed using one clinical isolate and one laboratory-adapted ATCC strain. Therefore, although the wound model revealed a clear difference between the two strains tested, additional clinical isolates will be required to determine whether this strain-dependent phenotype extends to a broader range of clinically derived *M. abscessus* strains. Second, the proposed host-cell-associated dissemination mechanism was inferred from splenic bacterial recovery and histopathological findings but was not directly tested by macrophage tracking, macrophage depletion, lineage tracing, or intracellular survival assays. Third, although a standardized inoculum was used in the current study, future dose-response analyses would further define the sensitivity and dynamic range of this model. Fourth, local and systemic cytokine profiles were assessed cross-sectionally using separate animal cohorts at each terminal time point; consequently, the temporal interpretations proposed here—including the suggested early IL-17A/IL-1β neutrophilic phase preceding a later IL-12/IFN-γ Th1 phase—were not directly tested statistically and should be confirmed by longitudinal sampling within individual animals together with the mechanistic perturbation experiments outlined above. Fifth, immune cell phenotyping was not performed in the present study. Although IFN-γ IHC and cytokine profiling support a Th1-associated immune response, the specific cellular sources and immune-cell composition of the granulomatous lesions remain to be defined. Future studies using flow cytometry, multiplex immunostaining, or spatial immune profiling will be required to identify macrophage subsets, neutrophils, T-cell populations, and antigen-presenting cells associated with lesion organization and bacterial persistence. These limitations should be considered when interpreting the wound model as a promising but not yet fully validated platform for evaluating strain-dependent pathogenic phenotypes.

## Conclusions

This study establishes a murine excisional wound infection model that recapitulates key pathological and immunological features of cutaneous *M. abscessus* infection. Compared with conventional subcutaneous injection, the wound model generated persistent pyogranulomatous lesions associated with sustained Th1-skewed immune responses, thereby more closely reflecting features observed in human cutaneous NTM disease. In the strain set examined here, comparison of one clinical isolate with ATCC 19977 suggests that the model may reveal strain-dependent differences in infection establishment. However, validation with additional clinical isolates is required before broader application of this model for evaluating strain-dependent pathogenic phenotypes. Overall, the excisional wound model provides a useful preclinical framework for investigating cutaneous *M. abscessus* pathogenesis and for future studies aimed at identifying bacterial and host determinants of persistent infection.

## Data Availability

The original contributions presented in the study are included in the article/**Supplementary Material**. Further inquiries can be directed to the corresponding author.
